# Supplementary material for: Transcriptome-wide mapping of small ribosomal subunits elucidates scanning mechanisms of translation initiation in the mammalian brain
Source: Commun Biol. 2025 Sep 30;8:1399. doi: 10.1038/s42003-025-08804-3 (PMC12484607; doi:10.1038/s42003-025-08804-3)
Supplement: Supplementary file 1 — Supplementary Information [file 42003_2025_8804_MOESM1_ESM.docx]

**Supplementary figures and tables**

**Transcriptome-wide mapping of small ribosomal subunits elucidates scanning mechanisms of translation initiation in the mammalian brain**

Preeti M Kute^1,2, ✝^, Francois P Pauzin^3, ✝^, Kornel Labun^1, ✝^, Clive R Bramham^3,4, *^*,* Eivind Valen^1,2,5, *^

1. Computational Biology Unit, Department of Informatics, University of Bergen, Bergen, Norway
2. Michael Sars Centre, University of Bergen, Bergen, Norway
3. Department of Biomedicine, Jonas Lies vei 91, University of Bergen, Bergen, Norway
4. Mohn Research Center for the Brain, University of Bergen, Bergen, Norway
5. Department of Biosciences, University of Oslo, Oslo, Norway

^✝^ These authors contributed equally

^*^ Corresponding authors: clive.bramham@uib.no, eivind.valen@ibv.uio.no

###
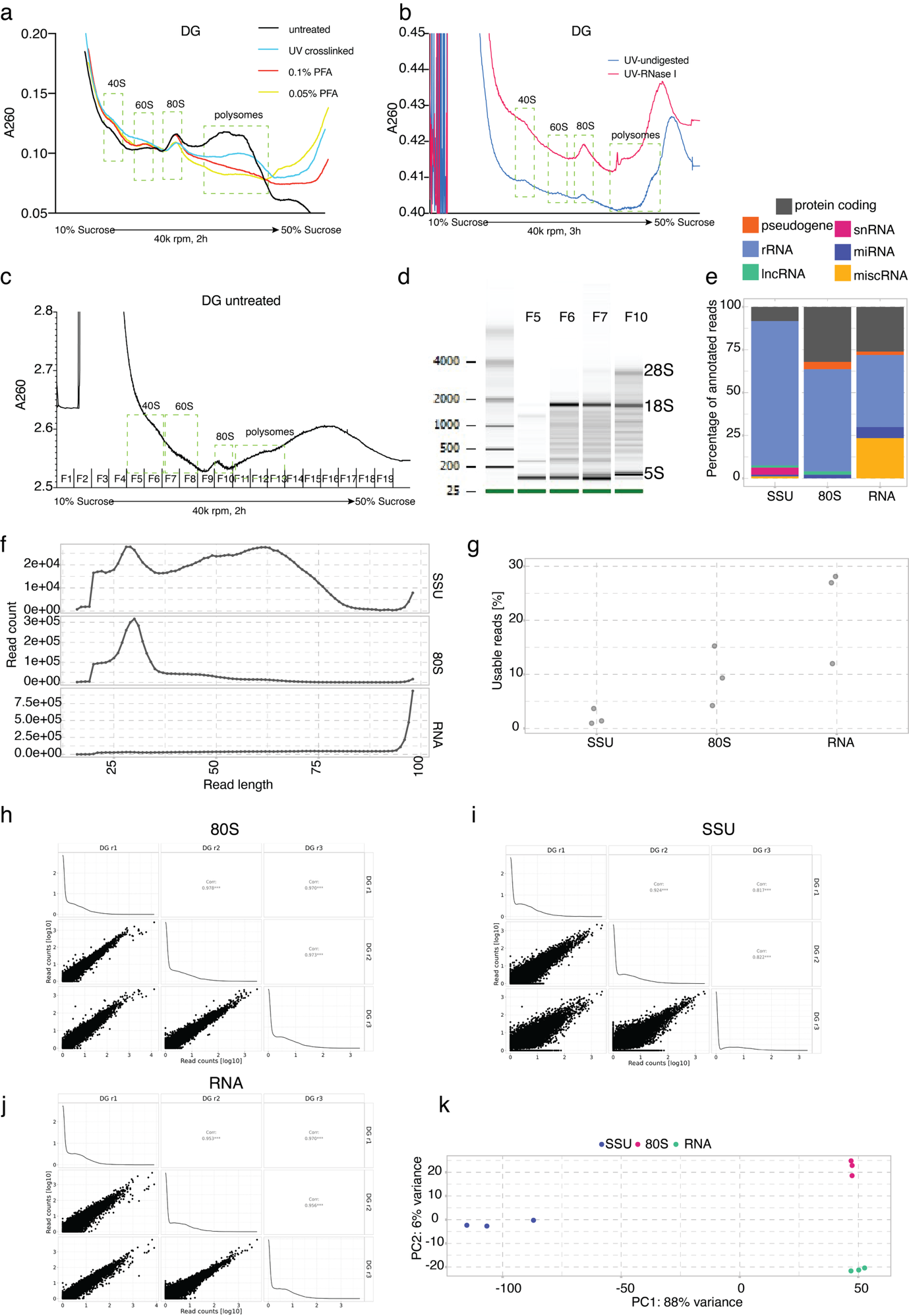


#### **Supplementary Figure 1: Quality assessment of the UV-crosslinking method for RCP-seq (Related to Figure 1)**

**a**) Amount of crosslinking for polysomes for three different cross-linking methods: formaldehyde, UV or non-crosslinked DG samples. **b**) Polysome profiles of UV-crosslinked, with or without RNase I digestion to denote increase in 80S for digested samples for DG. **c**) A UV trace of DG polysomes treated only with cycloheximide (no crosslinking) from a 10-15% sucrose gradient. 20 fractions were collected. Ribosomal subunits, 80S and polysomes are indicated. **d**) Bioanalyzer image of total RNA isolated from fractions from the above profile. From this image, fractions 5 and 6 contain 40S. **e**) Percent content displaying the mapping of SSU, 80S and total RNA counts to the type of transcript. **f**) Percent reads that are used for the gene expression analysis for SSU, 80S and RNA libraries. **g**) Footprint length distribution for the DG for SSU, 80S and RNA libraries. **h**) Pearson’s correlation for 80S among replicates in DG. **i**) Pearson’s correlation for SSU among replicates in DG. **j**) Pearson’s correlation for RNA among replicates in DG. **k**) PCA plot for SSU, 80S and RNA libraries from DG

####
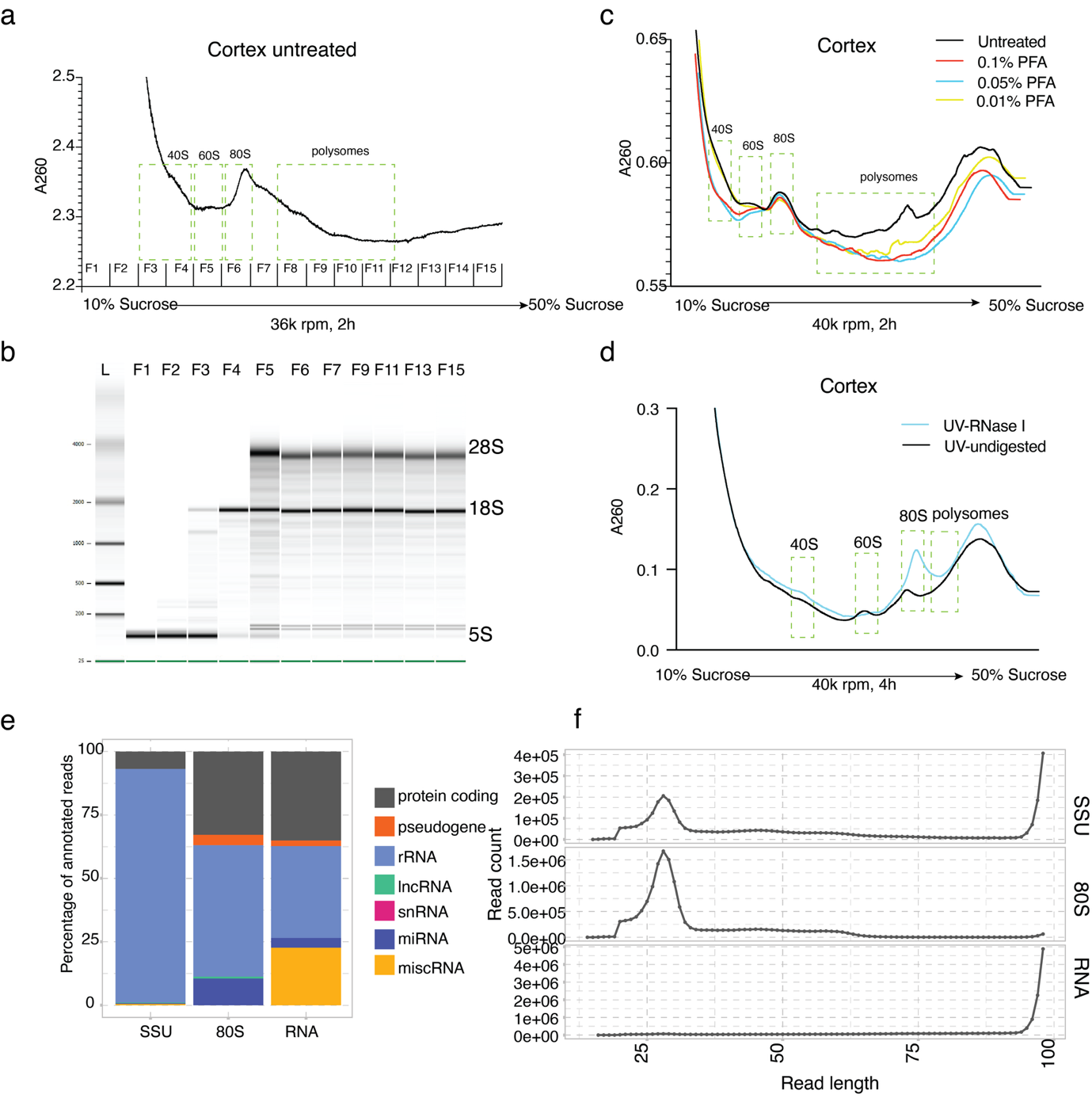
**Supplementary Figure 2: UV-crosslinking method for RCP-seq in the cortical tissue (related to figure 2)**

**a**) A UV trace of cortical polysomes treated only with cycloheximide (no crosslinking) from a 10-50% sucrose gradient. 20 fractions were collected out of which 15 fractions are shown. **b**) Bioanalyzer image of total RNA isolated from fractions from the above profile. From this image, fractions 3 and 4 may contain 40S. **c**) Amount of crosslinking for polysomes for three different paraformaldehyde concentrations vs non-crosslinked. **d)** Polysome profiles of UV-crosslinked, with or without RNase I digestion to denote an increase in the 80S for digested samples for cortical tissue. **e**) Percent content displaying the mapping of SSU, 80S and total RNA counts to the type of transcript. **f)** Footprint length distribution for the DG for SSU, 80S and RNA libraries.


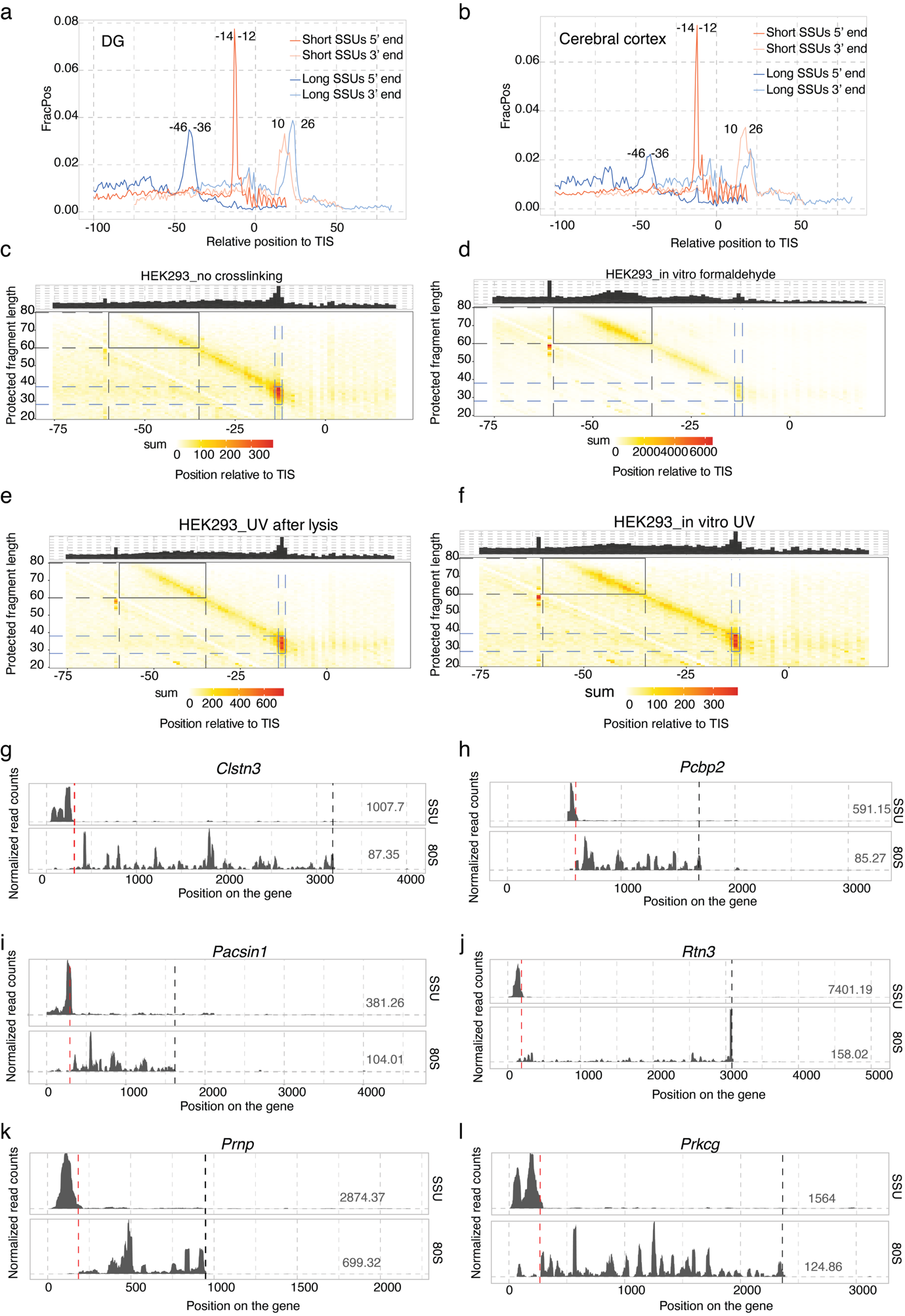


#### **Supplementary Figure 3: Estimation of SSU poising in 5’ leaders of transcripts from the DG and the cerebral cortex (Related to Figure 3)**

**a)** and **b)** Frequency distribution of SSU footprints based on their 5’ end position on the leaders **a**) for the DG and **b**) for the cerebral cortex. Numbers on the plots indicate nucleotide positions on the leaders relative to the TIS/start codon. **c**-**f** Footprint length distribution for the 5’ end of SSU fragments in HEK293T cells relative to the TIS, highlighting initiating SSUs (at -12nt) and poised SSUs (-36: -60nt) for different crosslinking condition, **c**) for non-crosslinked, **d**) for formaldehyde crosslinked, **e**) for UV crosslinked after lysis, **f**) for in vitro UV crosslinking. **g-l**: Single gene profile from DG for SSU and 80S, without intronic regions, and plotted for the longest isoform of *Clstn3* (Calsyntenin3, **g**), *Pcbp2* (Poly (rC) binding protein 2, **h**), *Pacsin1* (Protein Kinase C And Casein Kinase Substrate In Neurons 1, **i**), *Rtn3* (Reticulon 3, **j**), *Prnp* (Prion protein, **k**), *Prkcg* (Protein kinase C, gamma, **l**). Numbers in the plot indicate FPKM values. Dotted lines indicate TIS (red) and TTS (black).


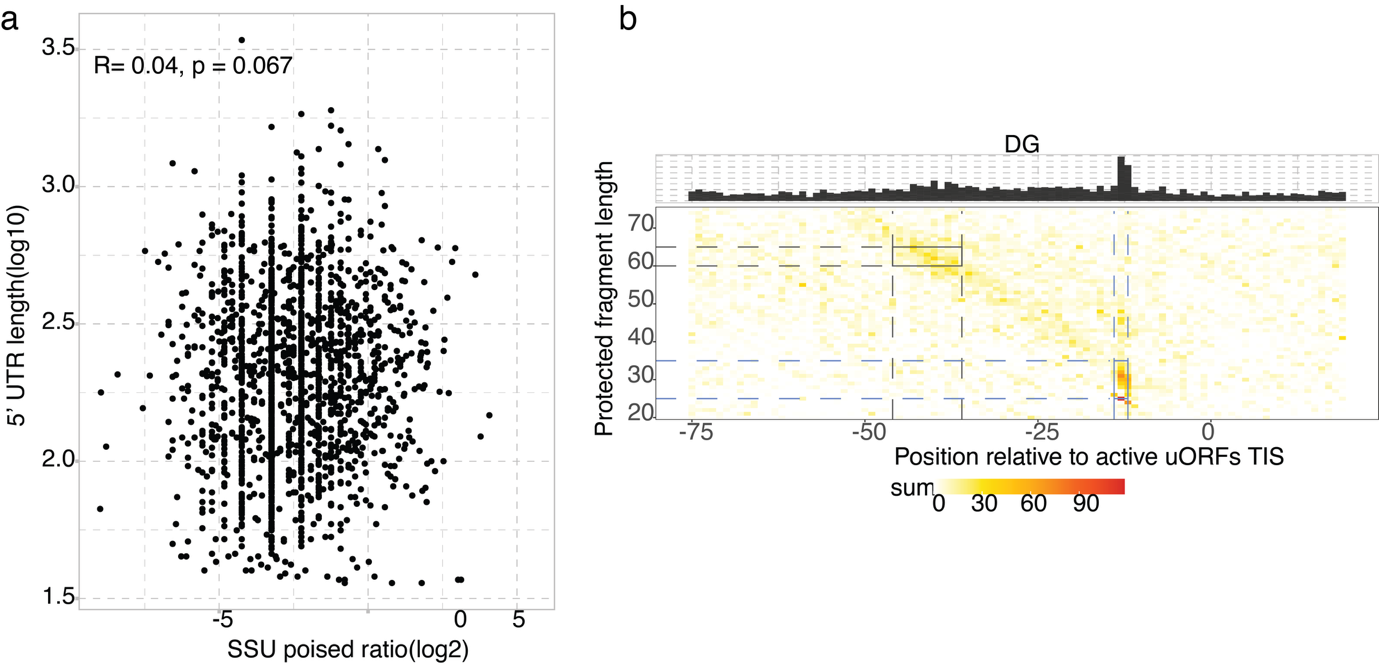


#### **Supplementary Figure 4: Effect of leaders on SSU poising (related to figure 4)**

**a**) Log2 of SSU poised ratio *vs*. the length of the 5’ UTRs for transcripts from DG. **b**) Footprint length distribution for the 5’ end SSU fragments relative to TIS of active uORFs in the DG tissue, highlighting initiating SSUs (at -12nt) and poised SSUs (-46: -42nt).

**Supplementary Table 1:** overview of descriptions of queued SSU

| **Reference** | **Model system** | **Localization** | **Suggested mechanism?** | **Detection method** |
| --- | --- | --- | --- | --- |
| ^1^ | HEK293T cells, reporter assays | SSU queuing on uORF, upstream of paused ribosome | high polyamines, elongation pausing (over PPW motif) during translation of the uORF | Model |
| ^2^ | HeLa cells, reporter assays | SSU queuing on ORF, upstream of paused ribosome | non-AUG start codons, slowed elongating ribosomes | Model |
| ^3^ | Yeast | SSU queuing on leaders with 5’ end -30nt rel to TIS | local structural or sequence features of mRNAs | TCP-seq |
| ^4^ | Yeast and HEK293T | In yeast, SSU queuing on leaders -30nt rel to TIS, for HEK293T SSU queuing on leaders -60 to -30nt rel to TIS |  | Sel-TCP seq and TCP-seq |
| ^5^ | NIH 3T3 | SSU queuing on leaders -120nt to -60nt rel to TIS | 80S ribosomes stalled at >12 codons downstream of TIS | Harringtonine treatment and TCP-seq |

**Supplementary data 1:** The source data behind the graphs (supplementary figures 1a-1c, supplementary figures 2a, 2c and 2d )

**Supplementary data 2:** Sequencing depth, contaminants mapping

**Supplementary data 3:** List of SSU poised-up genes in both DG and cortex

**Supplementary data 4:** Active uORFs discovered in the study in both DG and cortex

**References**

1. Ivanov, I. P. et al. Polyamine control of translation elongation regulates start site selection on antizyme inhibitor mRNA via ribosome queuing. Mol. Cell 70, 254-264.e6 (2018).

2. Kearse, M. G. et al. Ribosome queuing enables non-AUG translation to be resistant to multiple protein synthesis inhibitors. Genes Dev. 33, 871–885 (2019).

3. Archer, S. K., Shirokikh, N. E., Beilharz, T. H. & Preiss, T. Dynamics of ribosome scanning and recycling revealed by translation complex profiling. Nature 535, 570–574 (2016).

4. Wagner, S. et al. Selective Translation Complex Profiling Reveals Staged Initiation and Co-translational Assembly of Initiation Factor Complexes. Mol. Cell 79, 546-560.e7 (2020).

5. Bohlen, J., Fenzl, K., Kramer, G., Bukau, B. & Teleman, A. A. Selective 40S Footprinting Reveals Cap-Tethered Ribosome Scanning in Human Cells. Mol. Cell 79, 561-574.e5 (2020).
