# Supplementary material for: Transcriptome-wide mapping of small ribosomal subunits elucidates scanning mechanisms of translation initiation in the mammalian brain
Source: Commun Biol. 2025 Sep 30;8:1399. doi: 10.1038/s42003-025-08804-3 (PMC12484607; doi:10.1038/s42003-025-08804-3)
Supplement: Supplementary file 2 — Description of Additional Supplementary Materials [file 42003_2025_8804_MOESM2_ESM.pdf]

Description of Additional Supplementary Files

File name: Supplementary Data 1

Description: The source data behind the graphs (supplementary figures 1a-1c, supplementary figures 2a, 2c and 2d)

File name: Supplementary Data 2

Description: Sequencing depth, contaminants mapping

File name: Supplementary Data 3

Description: List of SSU poised-up genes in both DG and cortex

File name: Supplementary Data 4

Description: Active uORFs discovered in the study in both DG and cortex
