# Supplementary material for: Transcriptome-wide mapping of small ribosomal subunits elucidates scanning mechanisms of translation initiation in the mammalian brain
Source: Commun Biol. 2025 Sep 30;8:1399. doi: 10.1038/s42003-025-08804-3 (PMC12484607; doi:10.1038/s42003-025-08804-3)
Supplement: Supplementary file 7 — Reporting Summary [file 42003_2025_8804_MOESM7_ESM.pdf]

nature portfolio

Corresponding author(s): Prof. Eivind Valen and Prof. Clive Bramham

Last updated by author(s): Aug 23, 2025

Reporting Summary

Nature Portfolio wishes to improve the reproducibility of the work that we publish. This form provides structure for consistency and transparency in reporting. For further information on Nature Portfolio policies, see our Editorial Policies and the Editorial Policy Checklist.

Statistics

For all statistical analyses, confirm that the following items are present in the figure legend, table legend, main text, or Methods section.

n/a	Confirmed	
☐	☒	The exact sample size (*n*) for each experimental group/condition, given as a discrete number and unit of measurement
☐	☒	A statement on whether measurements were taken from distinct samples or whether the same sample was measured repeatedly
☐	☒	The statistical test(s) used AND whether they are one- or two-sided<br>*Only common tests should be described solely by name; describe more complex techniques in the Methods section.*
☒	☐	A description of all covariates tested
☐	☒	A description of any assumptions or corrections, such as tests of normality and adjustment for multiple comparisons
☐	☒	A full description of the statistical parameters including central tendency (e.g. means) or other basic estimates (e.g. regression coefficient) AND variation (e.g. standard deviation) or associated estimates of uncertainty (e.g. confidence intervals)
☐	☒	For null hypothesis testing, the test statistic (e.g. *F*, *t*, *r*) with confidence intervals, effect sizes, degrees of freedom and *P* value noted<br>*Give P values as exact values whenever suitable.*
☒	☐	For Bayesian analysis, information on the choice of priors and Markov chain Monte Carlo settings
☒	☐	For hierarchical and complex designs, identification of the appropriate level for tests and full reporting of outcomes
☒	☐	Estimates of effect sizes (e.g. Cohen's *d*, Pearson's *r*), indicating how they were calculated

Our web collection on statistics for biologists contains articles on many of the points above.

Software and code

Policy information about availability of computer code

Data collection	The repository at https://git.app.uib.no/valenlab/brain_dg_cortex_rcp and https://git.app.uib.no/valenlab/preeti_40s_2025 contains all the code that was used for analysis and all of the processed data and figures are available there for inspection.
Data analysis	The repository at https://git.app.uib.no/valenlab/brain_dg_cortex_rcp and https://git.app.uib.no/valenlab/preeti_40s_2025 contains all the code that was used for analysis. Statistical analysis was done using R and ggpubr package

For manuscripts utilizing custom algorithms or software that are central to the research but not yet described in published literature, software must be made available to editors and reviewers. We strongly encourage code deposition in a community repository (e.g. GitHub). See the Nature Portfolio guidelines for submitting code & software for further information.

Data

Policy information about availability of data

All manuscripts must include a data availability statement. This statement should provide the following information, where applicable:

- Accession codes, unique identifiers, or web links for publicly available datasets
- A description of any restrictions on data availability
- For clinical datasets or third party data, please ensure that the statement adheres to our policy

Supplementary Data 1-4 are available as supplementary data files. Sequencing raw reads are published under accession number PRJEB72224 in the European Nucleotide Archive.

Research involving human participants, their data, or biological material

Policy information about studies with human participants or human data. See also policy information about sex, gender (identity/presentation), and sexual orientation and race, ethnicity and racism.

Reporting on sex and gender	NA
Reporting on race, ethnicity, or other socially relevant groupings	NA
Population characteristics	NA
Recruitment	NA
Ethics oversight	NA

Note that full information on the approval of the study protocol must also be provided in the manuscript.

Field-specific reporting

Please select the one below that is the best fit for your research. If you are not sure, read the appropriate sections before making your selection.

☒ Life sciences ☐ Behavioural & social sciences ☐ Ecological, evolutionary & environmental sciences

For a reference copy of the document with all sections, see nature.com/documents/nr-reporting-summary-flat.pdf

Life sciences study design

All studies must disclose on these points even when the disclosure is negative.

Sample size	For each biological replicate, 1 cortical hemisphere (left/right) (~17ug RNA) was used. Thus, three biological replicate experiments were performed from cortical hemispheres (left/right) of 3 different mice. For DG tissue, 10 DGs (left + right) (~9ug RNA) from 5 mice were pooled for one biological replicate. Thus, three biological replicates were performed from 15 different mice.
Data exclusions	No data were excluded from analysis
Replication	Reproducibility of biological replicates was confirmed by Principal Component Analysis (PCA) and Pearson correlation analysis
Randomization	Not relevant. In this study all male WT offspring were taken into data
Blinding	Not relevant. In this study, we only used one group of WT mice.

Reporting for specific materials, systems and methods

We require information from authors about some types of materials, experimental systems and methods used in many studies. Here, indicate whether each material, system or method listed is relevant to your study. If you are not sure if a list item applies to your research, read the appropriate section before selecting a response.

Materials & experimental systems

n/a	Involved in the study	
☒	☐	Antibodies
☐	☒	Eukaryotic cell lines
☒	☐	Palaeontology and archaeology
☐	☒	Animals and other organisms
☒	☐	Clinical data
☒	☐	Dual use research of concern
☒	☐	Plants

Methods

n/a	Involved in the study	
☒	☐	ChIP-seq
☒	☐	Flow cytometry
☒	☐	MRI-based neuroimaging

Eukaryotic cell lines

Policy information about cell lines and Sex and Gender in Research

Cell line source(s)	HEK293T cells
Authentication	The cell line was not authenticated
Mycoplasma contamination	The cell line were tested for mycoplasma contamination using DAPI staining. No contamination was detected.
Commonly misidentified lines (See ICLAC register)	NA

Animals and other research organisms

Policy information about studies involving animals; ARRIVE guidelines recommended for reporting animal research, and Sex and Gender in Research

Laboratory animals	12 to 14-week-old C57BL/6 wild-type male mice were used. Mice were bred and housed in their home cages. Room temperature (22∘C (±1∘C)) and relative humidity (46±5%) were maintained. Mice had free access to water and food and were maintained on a 12-hour light/dark cycle.
Wild animals	NA
Reporting on sex	Only male mice were used in this study
Field-collected samples	NA
Ethics oversight	The research was approved by the Norwegian National Research Ethics Committee in compliance with EU Directive 2010/63/EU, ARRIVE guidelines.

Note that full information on the approval of the study protocol must also be provided in the manuscript.

Plants

Seed stocks	NA
Novel plant genotypes	NA
Authentication	NA
